# Cullin-4B E3 ubiquitin ligase mediates Apaf-1 ubiquitination to regulate caspase-9 activity

**DOI:** 10.1371/journal.pone.0219782

**Published:** 2019-07-22

**Authors:** Eri Ohta, Masanori Itoh, Masashi Ueda, Yoko Hida, Miao-xing Wang, Miki Hayakawa-Ogura, Shimo Li, Emika Nishida, Kazunori Ohta, Tana, Saiful Islam, Kiyomi Nakagawa, Tomomi Sunayama, Huayue Chen, So Hirata, Masashi Endo, Yoya Ohno, Toshiyuki Nakagawa

**Affiliations:** 1 Department of Neurobiology, Gifu University Graduate School of Medicine, Yanagido, Gifu, Japan; 2 Department of Anatomy School of Medicine, University of Occupational and Environmental Health, Iseigaoka, Fukuoka, Japan; Chung Shan Medical University, TAIWAN

## Abstract

Apoptotic protease-activating factor 1 (Apaf-1) is a component of apoptosome, which regulates caspase-9 activity. In addition to apoptosis, Apaf-1 plays critical roles in the intra-S-phase checkpoint; therefore, impaired expression of Apaf-1 has been demonstrated in chemotherapy-resistant malignant melanoma and nuclear translocation of Apaf-1 has represented a favorable prognosis of patients with non-small cell lung cancer. In contrast, increased levels of Apaf-1 protein are observed in the brain in Huntington’s disease. The regulation of Apaf-1 protein is not yet fully understood. In this study, we show that etoposide triggers the interaction of Apaf-1 with Cullin-4B, resulting in enhanced Apaf-1 ubiquitination. Ubiquitinated Apaf-1, which was degraded in healthy cells, binds p62 and forms aggregates in the cytosol. This complex of ubiquitinated Apaf-1 and p62 induces caspase-9 activation following MG132 treatment of HEK293T cells that stably express *bcl-xl*. These results show that ubiquitinated Apaf-1 may activate caspase-9 under conditions of proteasome impairment.

## Introduction

Apoptosis is an important mechanism in embryonic development and maintenance of tissue homeostasis[1]. Dysregulation of apoptosis is implicated in the pathogenesis of several diseases such as neurodegeneration and cancer. A central component of the apoptosis pathway is a family of cysteine proteases called caspases. Caspases are synthesized as inactive zymogens, and are activated by upstream caspases or by the formation of signaling complexes such as death-inducing signaling complex or DISC[2] and apoptosome[3]. The formation of apoptosome complex is critical for the initiation of apoptosis through caspase activation[4], and comprises homo-oligomers of apoptotic protease-activating factor 1 (Apaf-1) in mammals[3] or its homologs cell-death abnormal (CED)-4 in *Caenorhabditis elegans*[5] and *Drosophila* Apaf-1-related killer or Dark [also known as hac-1 (homolog of Apaf-1 and ced-4) or Dapaf-1 (*Drosophila* Apaf-1/ced-4 homolog)] in *Drosophila*[6–8]. Several factors regulate Apaf-1 action, which is enhanced by cytochrome *c* and dATP[3, 9], Parcs[10], and NAC[11], but inhibited by APIP[12], Aven[13], Hsp70[14, 15], and Hsp90[16] in mammals; in *C*. *elegans*, the translocation of CED-4 from mitochondria to the nuclear membrane, which is likely mediated by CED-4-interacting proteins such as matefin/SUN-1[17] and ribosomal protein L10a[18], promotes the activation of CED-3[19]; in *Drosophila*, the cytosolic protein fractions enhance Dark-mediated DEVD-like activity of Dronc[20].

Ubiquitin is a versatile molecule that plays a critical role in several cellular processes[21], including apoptosis where it is involved in the regulation of caspase activity[22]. The *Drosophila* inhibitor of apoptosis 1 (DIAP1) promotes the ubiquitination of Dronc[23, 24] and *Drosophila* ICE/CED-3-related protease[25], leading to the inactivation of caspases. Caspase activity is increased by UbcD1-mediated[26] and Hid-, Reaper-, and Grim-stimulated[27] ubiquitination and degradation of DIAP1 as well as Nutcracker, an F-box protein that controls proteasome activity during sperm differentiation[28, 29]. As in *Drosophila*, caspase-3[30–32], caspase-7[31, 32], caspase-8[33, 34], caspase-9[35, 36], and caspase-12[37] are ubiquitinated in mammals. Although ubiquitination of caspases generally results in their degradation and inactivation, the ubiquitination of caspase-9 is required for patched-induced apoptosis[36]. Moreover, Jin et al. demonstrated that the polyubiquitination of the C-terminal region of caspase-8 by Cullin-3 resulted in aggresome formation in the cytosol in a p62 (also known as sequestosome 1)-dependent manner, leading to caspase-8 activation[33]. p62 has several domains that interact with other proteins and regulate various cell signaling pathways; for instance, the ubiquitin-associated (UBA) domain of p62 binds to polyubiquitinated proteins[38, 39], which is required for aggregate formation, particularly under conditions of proteasome impairment.

Increased endogenous levels of Apaf-1 protein have been observed in human brain tumors, a consequence of E2F1 transcriptional activity[40], and in Huntington’s disease (HD) and its mouse and fly models[41]. These observations suggest that Apaf-1 protein levels in cells are subject to transcriptional as well as post-translational modulation. Because dysfunction of the ubiquitin-proteasome system (UPS) has been observed in HD[42], we hypothesize that the expression of Apaf-1 is regulated by UPS. In the present study, we investigated whether Apaf-1 is ubiquitinated and whether its ubiquitination affects caspase activity.

## Materials and methods

### Materials

The following antibodies and chemicals were purchased: anti- Cullin-4B, anti-FLAG (M2), and anti-α-tubulin antibodies, mouse IgG-agarose, monoclonal anti-HA-agarose, and anti-FLAG M2 affinity gel from Sigma-Aldrich Co. (St. Louis, MO); anti-caspase-3, anti-caspase-9, anti-cleaved caspase-3, and anti-cleaved caspase-7 antibodies from Cell Signaling Technology (Beverly, MA); anti-Apaf-1 antibody from Millipore (Temecula, CA) and Abcam (Cambridge, United Kingdom); anti-multi ubiquitin antibody (FK2) from Medical and Biological Laboratories Co., Ltd. (Nagoya, Japan); anti-p62 (C-terminal) antibody (specific to amino acids 421–440 of human p62 protein) from PROGEN Biotechnik GmbH (Heidelberg, Germany); anti-HA (HA.11 and Y-11) and anti-myc antibodies from Covance (Richmond, CA) and Santa Cruz Biotechnology (Santa Cruz, CA); and anti-ATF4, anti-REST, and anti-Bcl-xS/L antibodies from Santa Cruz Biotechnology. HRP-conjugated anti-rat, anti-mouse, anti-rabbit, and anti-guinea pig IgG (H + L) antibodies were purchased from Southern Biotech (Birmingham, AL); MG132 from Calbiochem (La Jolla, CA); etoposide, E64d, pepstatin A, and cycloheximide from Sigma-Aldrich Co. All other chemicals were purchased from Wako Pure Chemical Industries (Osaka, Japan), Kanto Chemical (Tokyo, Japan), and Sigma-Aldrich Co.

### DNA constructs

Dominant-negative *cullin-4B* (dnCul4B, 1–604 amino acid residues) fragment was generated from ORH09610 (Kazusa DNA Res. Inst., Chiba, Japan) using the restriction enzymes, ApaI and StuI, and subcloned into pBlueScript SK (Agilent Technologies, Inc., Santa Clara, CA). dnCul4B was subcloned as a *Kpn* I–*Pst* I fragment into pcDNA6/myc-HisB (Invitrogen) to generate dnCul4B-myc. Human *p62* cDNA was generated by reverse transcription (RT)-PCR of RNA harvested from HeLa cells using the oligonucleotides 5′-ctcgaattcctatggcgtcgctcaccgtgaag-3′ and 5′-gcaattctacgcaagcttaacacaactatgagac-3′. *p62* cDNA was subcloned into pEGFP-C3 and pECFP-C1 vectors (Clontech, Mountain View, CA) to generate GFP-*p62* and CFP-*p62*, respectively. *p62*-FLAG was generated by the amplification of *p62* cDNA using the oligonucleotides 5′-ctcgaattcctatggcgtcgctcaccgtgaag-3′ and 5′-ctcgtcgaccaacggcgggggatgctttgaatac-3′, followed by subcloning into pFLAG-CMV-5a (Sigma-Aldrich Co.). GFP-tagged del UBA *p62* was generated by subcloning *p62* cDNA lacking the C-terminal region (amino acid residues 389–440) into pEGFP-C3. Human *cullin-4B* cDNA was amplified by PCR using ORH09610 (Kazusa DNA Res. Inst., Chiba, Japan) as template and the oligonucleotides 5′-ctcaagcttatgatgtcacagtcatctggatc-3′ and 5′- ctcgtcgactatgcaatatagttgtactggtttgg-3′, and subcloned into pFLAG-CMV-2 (Sigma-Aldrich Co.) or pEGFP-C3 (Clontech). Human *ubiquitin* cDNA was amplified by RT-PCR using the oligonucleotides 5′-ctcgaattcgcagattttcgtgaaaacccttac-3′ and 5′-ctcggatccttaaccaccacgaagtctcaacac-3′, and subcloned into pFLAG-CMV-2 (Sigma-Aldrich Co.) or pECFP-C1 (Clontech). All lysine residues were substituted with arginine in *Ub*K0. *Ub*K0^G76A^ (glycine-to-alanine substitution at position 76) was generated by mutagenesis of *ubiquitin* K0 cDNA. *Ub*K0^G76A^ was subcloned into pcDNA3.1/HisB (Invitrogen) and pcDNA-HA. All sequences were confirmed by DNA sequencing. Nucleotide sequences of the primers for mutagenesis are shown (Table 1).

**Table 1:** Sequences of oligonucleotides used in this mutagenesis.

gene	position and mutagenesis	sequence-1	sequence-2
CED-3	C358S	5′-cgtttttgtgcaggcttCtcgaggcgaacgt-3′	5′-acgttcgcctcgaGaagcctgcacaaaaacg-3′
CED-4S	K212R	5′-tttacggatattttgctgatgctaaGaagcgaagacgatcttctcaa-3′	5′-ttgagaagatcgtcttcgcttCttagcatcagcaaaatatccgtaaa-3′
	K175R	5′-gcatcacaagctctttcgaGatctgaccaacttattgga-3′	5′-tccaataagttggtcagatCtcgaaagagcttgtgatgc-3′
	K191R	5′-gattcaatcgtttggctcaGagatagtggaacagct-3′	5′-agctgttccactatctCtgagccaaacgattgaatc-3′
	K198R	5′-caaagatagtggaacagctccaaGatctacattcgatttatttacg-3′	5′-cgtaaataaatcgaatgtagatCttggagctgttccactatctttg-3′
	K232R	5′-cacgtcagttgtactcaGaaggatgatctgcaacg-3′	5′-cgttgcagatcatccttCtgagtacaactgacgtg-3′
Apaf-1	K224R	5′-cttccacttaatattgaagaggctaGagaccgtctccgcattctgatgc-3′	5′-gcatcagaatgcggagacggtctCtagcctcttcaatattaagtggaag-3′
	K192R*	5′-cagttgggaaacaagacaGatctgggcttctgatgaa-3′	
	K252R*	5′-ttgggactcttgggtgttgaGagcttttgacagtc-3′	
	K637R*	5′-gcttcttgtggagctgataGaaccttacaggtgttca-3′	
Ubiquitin	G76A	5′-gagacttcgtggtgCttaaggatcccggg-3′	5′-cccgggatccttaaGcaccacgaagtctc-3′
	K6R	5′-ttcgcagattttcgtgaGaacccttacggggaag-3′	5′-cttccccgtaagggttCtcacgaaaatctgcgaa-3′
	K6,11R	5′-gaGaacccttacggggaGgaccatcaccctcgagg-3′	5′-cctcgagggtgatggtcCtccccgtaagggttCtc-3′
	K33R	5′-ggccaagatccaggataGggaaggaattcctcctg-3′	5′-caggaggaattccttccCtatcctggatcttggcc-3′
	K27,29,33R	5′-ctcggatacgatagaaaatgtaaGggccaGgatccaggataGg-3′	5′-cCtatcctggatcCtggccCttacattttctatcgtatccgag-3′
	K48R	5′-agagactgatctttgctggcaGgcagctggaaga-3′	5′-tcttccagctgcCtgccagcaaagatcagtctct-3′
	K63R	5′-acgtactttgtctgactacaatattcaaaGggagtctactcttca-3′	5′-tgaagagtagactccCtttgaatattgtagtcagacaaagtacgt-3′

Capital letter is a position of mutagenesis. Mutagenesis is carried out by using QuickChange II Site-Directed or QuickChange Multi Site-Directed (*) Mutagenesis Kits.

### siRNA and transfection

The following siRNA were used: *cullin-1*: 5′-AUAACUACGUUCCAACUCUGACGGC-3′; *cullin-2*: 5′-AUAAGCAGUCCAUAUAGUCUGCACC-3′; *cullin-3*: 5′-AAUAAUGAGAGGUAUUCAGGAGACC-3′; *cullin-4A*: 5′-UCAUGAUCUUGUCCAACGUCCGCUC-3′; *cullin-4B*: 5′-AAAGAACGCUAUCCAAUGAAUCCUC-3′; *cullin-5*: 5′-UAGCAAGCUUGUUUACAUAAUCCGC-3′; *cullin-7*: 5′-UGCAAAUACCGCUUCACCAGGGAGA-3′; Stealth RNAi Negative Control Duplexes (Invitrogen). HEK293T cells were transfected with 50 pmol of siRNA and *apaf-1*-HA using Lipofectamine 2000 (Invitrogen, Carlsbad, CA). The expression of *cullin* mRNAs was evaluated by real-time RT-PCR.

### Cell culture and generation of stable cell lines

HEK293T cells were grown in complete media containing DMEM (Wako) supplemented with 10% FBS and Penicillin-Streptomycin (Invitrogen). Plasmids were transfected using Lipofectamine 2000, 3000 (Invitrogen) or the calcium phosphate-DNA precipitation method. Cell viability was determined using CellTiter 96 Aqueous Non-Radioactive Cell Proliferation Assay or CellTiter-Gl Luminescent Cell Viability Assay (Promega, Madison, USA) according to the manufacturer’s protocol. For analysis of nuclear fragmentation, cells were fixed with 4% paraformaldehyde in 0.1 M phosphate buffer (pH 7.2), permeabilized using 0.4% Triton X-100 in phosphate-buffered saline, and stained with bis-benzamide (Sigma-Aldrich Co.). The percentage of nuclear fragmentation was calculated by counting nuclei in randomly selected photos. For generating knockdown cells, DNA oligonucleotides for hairpin RNA expression were inserted into pSuper vector, followed by co-transfection of cells with pSuper and pTK-Hyg (Clontech) plasmids and selection using 100–250 μg/ml hygromycin (Wako). The DNA oligonucleotides 5′-gattgtgaacatcttcaat-3′ or 5′-ggaaatcttcgtcttatga-3′ or 5′-gcattgaagttgatatcga-3′ containing the target sequence was used for generating *caspase-9* or *apaf-1* or *p62* knockdown cells, respectively. HEK293T cells, which stably expressed *bcl-xl*, were generated by co-transfection with Xpress tagged-mouse *bcl-xl* and pBabe-puro vector, followed by selection using 2 μg/ml puromycin. Quantitative reverse transcription PCR (RT-PCR) was performed using SYBR Green (Thermal Cycler Dice, Takara Bio, Japan) according to the manufacturer’s protocol.

### Microscopic analyses

Cells were fixed with 4% paraformaldehyde in 0.1 M phosphate buffer (pH 7.2) 48 h post-transfection and mounted using Fluoromount (Diagnostic BioSystems, Pleasanton, CA). To detect release of cytochrome *c* from mitochondria, wild type and *bcl-xl* stable HEK293T cells were treated with 100 μM etoposide for 32 h and then 25 μM MG132 for 4 h. After fixation and permeabilization by methanol at -20°C for 10 min, cells were blocked with 10% normal donkey serum, and labeled with mouse anti-cytochrome *c* (BD Biosciences, Franklin Lakes, NJ, lot number 7H8.2C12) and rabbit anti-COX IV (Cell Signaling Technology) antibodies at 4°C overnight. The next day, cells were stained with Cy3-conjugated anti-mouse IgG and FITC-conjugated anti-rabbit IgG antibodies (Jackson ImmunoResearch), containing 10 μg/ml bis-benzamide at 4°C for 30 min. Samples were examined by confocal microscopy (LSM510 or LSM710, Carl Zeiss, Germany).

### Co-immunoprecipitation assay

Cells were co-transfected with *apaf-1*-HA and *p62*-FLAG using Lipofectamine 2000 (Invitrogen) or *apaf-1*-HA together with FLAG-*p62* or FLAG-del UBA *p62* by Calcium phosphate-DNA co-precipitation method, and lysed in IP buffer A (20 mM HEPES-KOH [pH 7.5], 150 mM NaCl, 10 mM KCl, 1.5 mM MgCl_2_, 0.3% CHAPS, 1 mM EDTA, 1 mM EGTA, and 1 mM dithiothreitol) in the presence of 25 μM MG132 and protease inhibitors (100 μM phenylmethylsulfonyl fluoride, 2 μg/ml aprotinin, 1 μg/ml pepstatin A, and 2 μg/ml leupeptin). Lysates were pre-cleared with protein G beads (Zymed, San Francisco, CA) to minimize non-specific binding, and immunoprecipitation was performed using anti-HA antibody immobilized onto protein G beads. The beads were subsequently washed with IP buffer A. For the detection of ubiquitination of Apaf-1-HA, cells were lysed in SDS-lysis buffer (150 mM Tris-HCl [pH 7.6], 50 mM NaCl, 1% SDS, 25 μM MG132, and 10 mM N-ethylmaleimide) in the presence of protease inhibitors. The lysate was heated at 95°C for 10 min, followed by centrifugation. The supernatant (100 μl) was diluted with 1300 μl of IP lysis buffer B (50 mM Tris-HCl [pH 7.4], 150 mM NaCl, 1 mM EDTA, 1% Triton X-100, and 25 μM MG132) containing protease inhibitors, and immunoprecipitated with anti-FLAG M2 affinity gel or anti-HA-agarose antibody (Sigma-Aldrich Co.) at 4°C. After three washes each with IP lysis buffer B containing 150 mM NaCl followed by 500 mM NaCl, ubiquitinated proteins were eluted using 3X FLAG peptide (Sigma-Aldrich Co.) or SDS-sample buffer. For the detection of interactions between Apaf-1 and Cullin-4B, cells with or without 100 μM etoposide treatment for 32 h were employed, and immunoprecipitation was performed in IP buffer A containing protease inhibitors.

### Caspase activity assay

Cells were treated with 100 μM etoposide for 32 h or 25 μM MG132 for 4h after transfection, and then washed with cold PBS (−) and incubated in 50 μl of chilled reaction buffer (100 mM HEPES-KOH [pH 7.2], 0.1% CHAPS, 10% sucrose, and 10 mM dithiothreitol) supplemented with protease inhibitors for 30 min on ice. Floating cells were lysed by three freeze-thaw cycles. The lysate was centrifuged for 30 min at 16 000 *×g*, and the resulting clear supernatant was used for caspase activity assay. Samples (50 μg) were incubated with fluorogenic substrates (100 μM Ac-DEVD-AFC, 100 μM Ac-LEHD-AFC; Biomol, Plymouth Meeting, PA) for 30 min at 37°C. Caspase activity was determined with a fluorometer (Fluoroskan Ascent; Thermo Fisher Scientific, Waltham, MA) or 2104 EnVision multilabel plate readers (Perkin Elmer, Waltham, MA) using Caspase-Gl Assays (Promega) according to the manufacturer’s protocol.

### Chromatography

For the generation of infectious lentiviral particles, *apaf-1*-HA, HA-*ubiquitin*, or HA-*ubiquitin* K0^G76A^ cDNA were subcloned into CSII-CMV-MCS (RIKEN, Tsukuba, Japan), and transfected with pCAG-HIVgp and pCMV-VSV-G-RSV-Rev (RIKEN) into 293FT cells (Invitrogen) using the calcium phosphate-DNA precipitation method. At three days post-transfection, the virus-containing supernatant was collected and filtered through 0.45 μm filter, and stored at −80°C. *Apaf-1* lentiviral particles were transduced into HEK293T cells followed by treatments with 25 μM MG132 for 4 h. *Ubiquitin* (wild type or K0^G76A^ mutant) lentiviral particles were transduced into HEK293T *bcl-xl* stable cells followed by treatments with 100 μM etoposide for 32 h and 25 μM MG132 for 4 h.　Cells were lysed in the buffer (PIPES-KOH [pH 7.4], 0.1% CHAPS, 2 mM EDTA, and 5 mM dithiothreitol) containing protease inhibitors, and subjected to 5 freeze-thaw (dry ice—37°C water) cycles. Cell lysate was centrifuged at 800 *×g* for 10 min, and the resulting supernatant was further centrifuged at 100,000 *×g* for 1 h. The supernatant thus obtained was termed S-100, and 1.6 mg of S-100 was fractionated using Superose 6 10/300 GL column connected to an AKTAexplorer 900 chromatography system (GE Healthcare Bio-Sciences AB, Uppsala, Sweden) with a flow rate of 0.4 ml/min in a solution containing 5% sucrose, 0.1% CHAPS, 50 mM NaCl, 20 mM HEPES-NaOH (pH 7.4), and 5 mM dithiothreitol. The column was calibrated with a molecular weight standard (Bio-Rad Laboratories, Hercules, CA). Aliquots of 23 μl or 40 μl of the fractions were directly subjected to caspase activity assay using Caspase-Gl (Promega).

### Statistical analyses

KaleidaGraph software (Synergy Software) was employed for the determination of statistical significance of differences obtained by the unpaired, two-tailed Student’s *t*-tests or one-way ANOVA followed by Tukey or Dunnett post-hoc test when necessary. Graphs represent mean and independent data points from three independent experiments.

## Results

### The ubiquitination of Apaf-1 is enhanced by etoposide treatment

**Fig 1:**
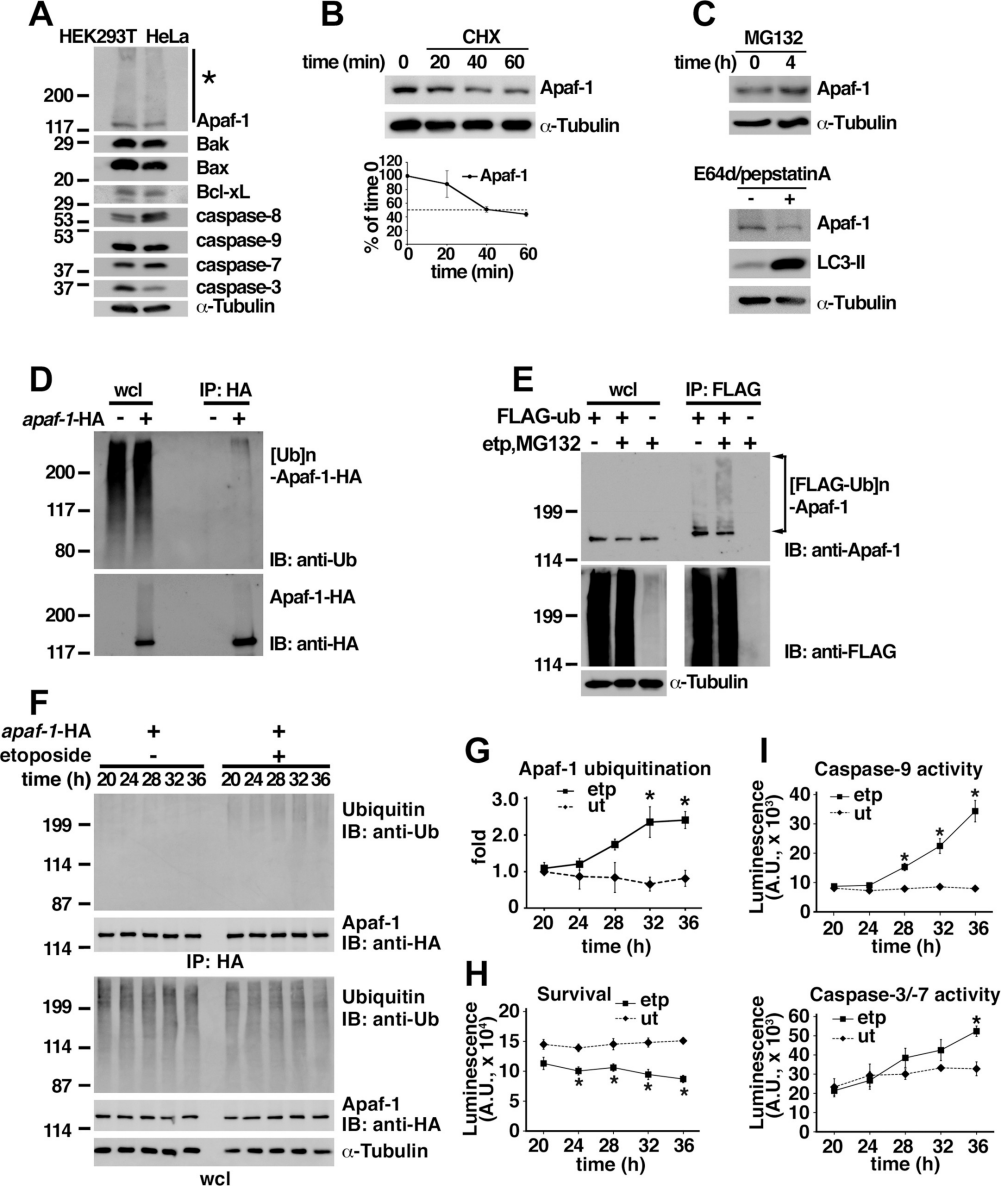
Ubiquitination of Apaf-1 is increased by etoposide treatment in HEK293T cells. (**A**) Slower-migrating species of Apaf-1 was detected by western blotting in HEK293T and HeLa cells (asterisk). (**B**) Half-life of Apaf-1 protein in HEK293T cells was examined by cycloheximide (CHX) treatment. The quantification of relative Apaf-1, corrected with respect to loading control, was determined by measuring the signal intensity using multi gauge ver3.0 software (Fujifilm). The dotted line denotes half-life. Graphs represent mean and independent data points from three independent experiments. (**C**) Levels of Apaf-1 protein in HEK293T cells were increased upon treatment with 25 μM MG132 for 4 h but not 10 μg/ml E64d along with 10 μg/ml Pepstatin A for 24 h. α-Tubulin was used as loading control. (**D, E**) Immunoprecipitation was performed with HEK293T cell extracts using anti-HA-agarose antibody after the transfection of *apaf-1*-HA (D) or anti-FLAG M2 affinity gel after the transfection of FLAG-*ubiquitin* (E). Endogenous Apaf-1 was ubiquitinated following treatment with etoposide for 36 h in HEK293T cells expressing FLAG-*ubiquitin* (E). IP: immunoprecipitation; IB: immunoblot; Ub: ubiquitin; anti-Ub: anti-multi ubiquitin antibody (FK2); wcl: whole cell lysate. (**F–I**) Immunoprecipitation using anti-HA antibody from HEK293T cells transfected with *apaf-1*-HA with or without etoposide treatment (F). The extent of Apaf-1 ubiquitination (fold of untreated at 20 h) was determined using ImageJ software (NIH, Bethesda, MD) after analysis of western blotting using ImageQuant LAS4000 (GE Healthcare Bio-Sciences AB) (G). Survival (H) as well as caspase-9 and caspase-3/-7 activities (I) was determined using CellTiter-Glo Luminescent Cell Viability Assay and Caspase-Glo Assays, respectively. time (H): hours after treatment with etoposide. *: *p < 0*.*005*. Graphs represent mean and independent data points from three independent experiments.

We observed the slower-migrating species of Apaf-1 (Fig 1A) in HeLa and human embryonic kidney (HEK) 293T cells by western blotting that prompted investigations of Apaf-1 modification by ubiquitination. Apaf-1 protein was rapidly degraded in HEK293T cells upon cycloheximide treatment (Fig 1B), while increased Apaf-1 protein levels were detected upon treatment with the proteasome inhibitor MG132 but not the autophagy inhibitors E64d and pepstatin A (Fig 1C). Immunoprecipitation experiments showed that transfected Apaf-1 was ubiquitinated with endogenous ubiquitin in HEK293T cells (Fig 1D). The ubiquitination of endogenous Apaf-1 increased after etoposide treatment (Fig 1E). Although FLAG-tagged *ubiquitin* (where the tag contains lysine residues) was employed for the experiment, Apaf-1 ubiquitination was also observed using HA-tagged *ubiquitin*, where the tag lacks lysine residues (S1A Fig); therefore, FLAG-tagged *ubiquitin* was used for subsequent experiments. Consistently, ubiquitination of Apaf-1 showed significant increase in a time-dependent manner following treatment with etoposide (Fig 1F and 1G), which correlates with decrease of cell survival (Fig 1H) and increase of caspase-9 and caspase-3/-7 activation (Fig 1I). To examine whether Apaf-1 homologs are similarly ubiquitinated, HEK293T cells were transfected with *dapaf-1* or *ced-4*, and immunoprecipitation was carried out. Ubiquitination of Dapaf-1 and CED-4 was observed in mammalian cells, particularly upon treatment with MG132 for 4 h (S1B and S1C Fig). The lysine at position 224 of human Apaf-1 (S2A Fig) has been recently identified as a ubiquitination site of Apaf-1 in bortezomib-treated cells by systematic screening[43]. Since this lysine is close to that of Lys-212 in CED-4, we investigated the ubiquitination after lysine-to-arginine substitutions were generated in Apaf-1 and CED4 (S2B Fig). Ubiquitination was slightly, but significantly decreased in CED4 (S1D Fig) and Apaf-1 (S1E Fig) mutants.

### Cullin-4B is the E3 ubiquitin ligase for Apaf-1

**Fig 2:**
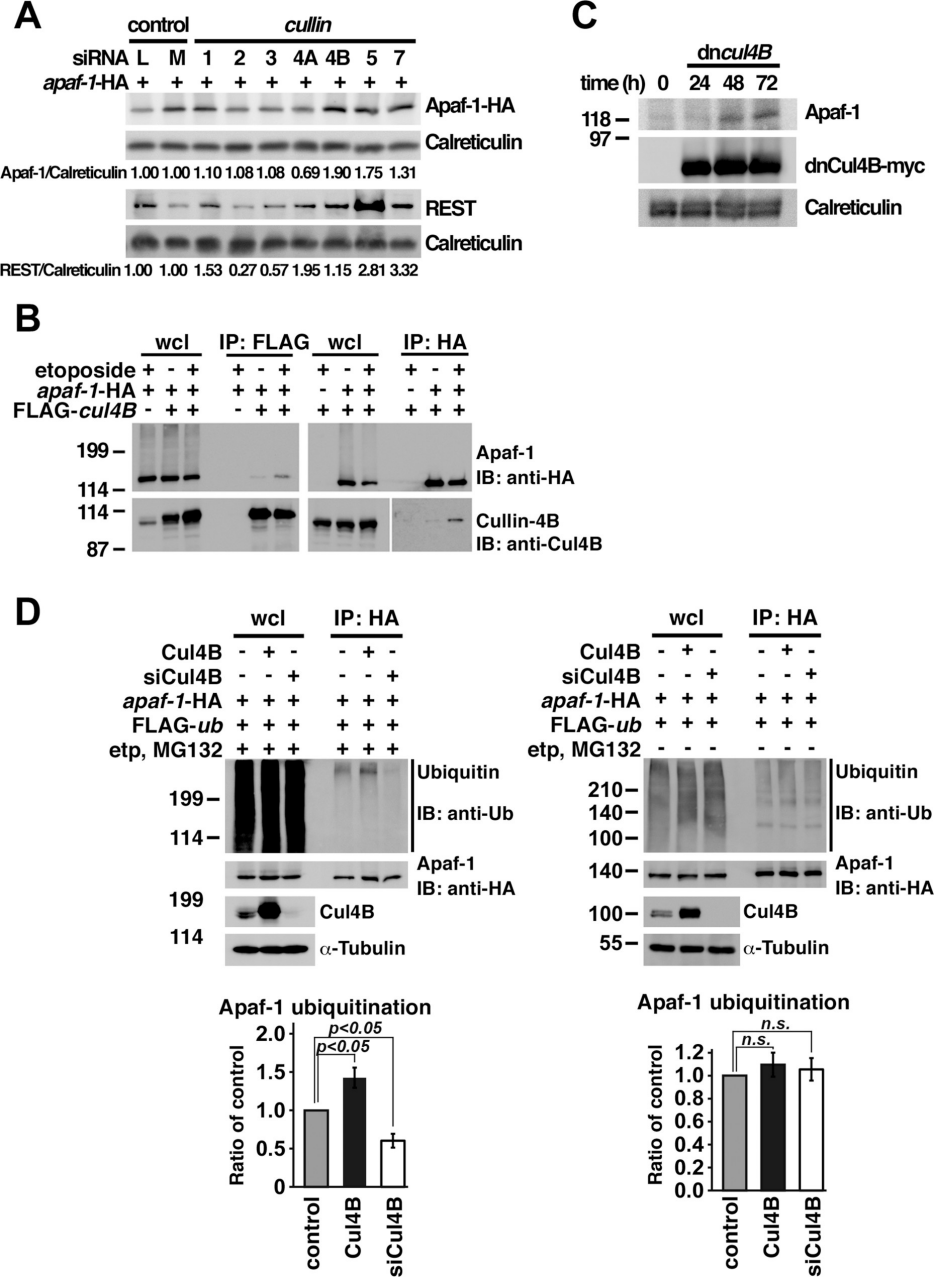
Cullin-4B is the E3 ubiquitin ligase for Apaf-1. (**A**) Increased Apaf-1-HA expression was seen in HEK293T cells expressing siRNA for *cullin-4B*. REST is ubiquitinated by Cullin-1 E3 ligase complex that was increased in *cullin-1*-knocked down cells. Calreticulin was used as loading control. (**B**) Endogenous Apaf-1 expression was increased in HEK293T cells expressing dominant-negative *cullin-4B* (dnCul4B). Calreticulin was used as loading control. (**C**) Interaction of Apaf-1 with Cullin-4B was enhanced with etoposide treatment. (**D**) Apaf-1 ubiquitination showed increase in *cullin-4B*-expressing but not *cullin-4B* knockdown cells in response to apoptotic stimulation (left), but not changed in untreated cells (right). Upper panels: western blotting; lower panel: levels of Apaf-1 ubiquitination from three independent experiments. siCul4B: siRNA for *cullin-4B*. Graphs represent mean and independent data points from three independent experiments.

Previously, Westbrook et al. knocked down β-TRCP1 and showed that REST (RE1-Silencing Transcription factor) was increased in these cells, to prove β-TRCP1 function as an E3 ligase to degrade REST[44]. According to this strategy, HEK293T cells were transfected with small interfering RNA (siRNA) for *cullin-1*, *-2*, *-3*, *-4A*, *-4B*, *-5*, and *-7*, and the levels of Apaf-1 protein were examined to identify the E3 ubiquitin ligase of Apaf-1. Increased levels of transfected and endogenous Apaf-1 were observed in *cullin-4B* knockdown cells (Fig 2A) and in HEK293T cells expressing Cullin-4B with C-terminal truncation (Cul4B_1-604_, or dnCul4B; Fig 2B), respectively. Because Cul4B_1-604_ likely inhibits endogenous Cullin-4B and Cullin-4B-like proteins[44], the binding of Apaf-1 to Cullin-4B was subsequently examined. Immunoprecipitation experiments showed that the interaction of Apaf-1 with Cullin-4B was enhanced upon etoposide treatment (Fig 2C). Moreover, Apaf-1 ubiquitination was observed to increase in HEK293T cells overexpressing *cullin-4B*, but decreased in *cullin-4B* knockdown HEK293T cells (Fig 2D, left). When cells were not treated with etoposide and MG-132, ubiquitination of Apaf-1 was not different among Cullin-4B endogenous, overexpressed, or knocked down cells (Fig 2D, right). Furthermore, even though *cullin-4B* was knocked down, Apaf-1 was ubiquitinated in untreated cells (S1F Fig). It might represent that Apaf-1 was ubiquitinated in physiological condition not by Cullin-4B, although E3 ligase, such as Cullin-5 and -7, have not been yet identified. These results suggest that Cullin-4B is likely to be the E3 ubiquitin ligase for Apaf-1.

### Ubiquitinated Apaf-1 interacts with p62

**Fig 3:**
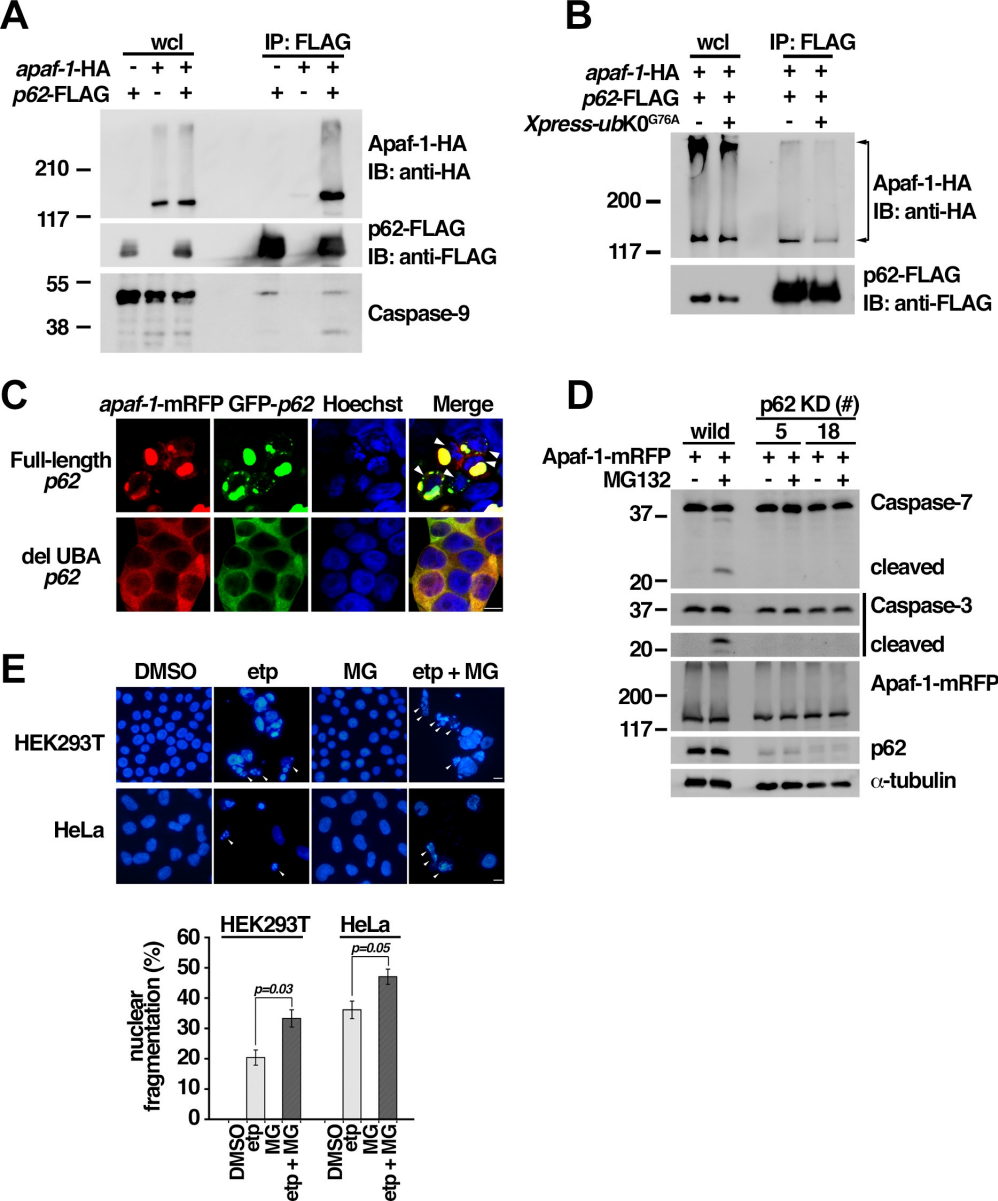
Apaf-1 interacts with p62. (**A**, **B**) Co-immunoprecipitation was performed following the transfection of *apaf-1*-HA and *p62*-FLAG into HEK293T cells. Apaf-1 interacted with p62 (A). Interaction of Apaf-1 with p62 showed a significant reduction in the presence of dominant-negative ubiquitin mutant *ub*K0^G76A^ (B). (**C**) Nuclear fragmentation was observed in cells expressing Apaf-1 with full-length p62 but not UBA domain-deleted p62 (del UBA p62). Nuclear fragmentation was estimated by counting from randomly selected images of Hoechst staining obtained using fluorescent microscopy. (**D**) The activation of caspase-3 and caspase-7 was not observed upon MG132 treatment in *p62* knockdown cells. (**E**) MG132 enhances etoposide-induced cell death. Representative photograph of Hoechst staining (left panels). Arrowhead: nuclear fragmentation. Percentage cell death (evaluated by nuclear fragmentation) in HEK293T and HeLa cells. Treatments include etp: 100 μM etoposide for 40 h; MG: 25 μM MG132 for 4 h; etp + MG: 25 μM MG132 was added 36 h after the addition of 100 μM etoposide for the total treatment of 40 h. Graph represents mean and independent data points from three independent experiments. (C, E): representative photograph of confocal microscope images. Scale bar: 10 μm.

Since p62 is involved in the formation of aggregates containing ubiquitinated proteins under conditions of proteasome impairment[45] and in autophagy-deficient mice[46], we examined the interaction of Apaf-1 and p62. Co-immunoprecipitation revealed the interaction of p62 with Apaf-1 (Fig 3A), which was decreased to a significant extent in the presence of dominant-negative *ubiquitin*[47] (UbK0^G76A^) (Fig 3B). Apaf-1 was co-localized with p62, forming aggregates in the cytosol (Fig 3C and S3A Fig), particularly upon MG132 treatment (S3B Fig). The role of the ubiquitin-associated (UBA) domain of p62 in the formation of Apaf-1 aggregates was also examined, because UBA is critical for the interaction of p62 with ubiquitinated proteins[39]; Apaf-1 failed to show aggregate formation with UBA domain-deleted p62 (del UBA p62; Fig 3C). A percentage of nuclear fragmentation was increased in cells with aggregates of Apaf-1 and p62 (30.2%, cell numbers = 86) but not in cells expressing del UBA p62 (3.9%, cell numbers = 178) (Fig 3C). Moreover, large fragments detected by antibodies specific to the cleaved products of caspase-3 and -7 were not observed by western blotting in p62-knockdown HEK293T cells following MG132 treatment (Fig 3D), suggesting that MG132 may induce apoptosis in the presence of Apaf-1 and p62 complex. We revealed by immunoprecipitation that Apaf-1 interacted with not only full length but also del UBA p62 (S3C Fig). However, del UBA p62 induced less caspase-9 activation than full length p62 (S3D Fig). Therefore, not just interaction but aggregation formation would be important for caspase-9 activation. Since etoposide enhanced the Apaf-1 ubiquitination through the interaction of Apaf-1 and Cullin-4B (Figs 1F and 2C) and caspase activities (Fig 1I), we examined whether the cell death was affected by MG132 after etoposide treatment. Treatment with MG132 for 4 h resulted in significantly enhanced etoposide-induced cell death in HEK293T and HeLa cells (Fig 3E).

### MG132 promotes caspase-9 activation in cells that stably express *bcl-xl*

**Fig 4:**
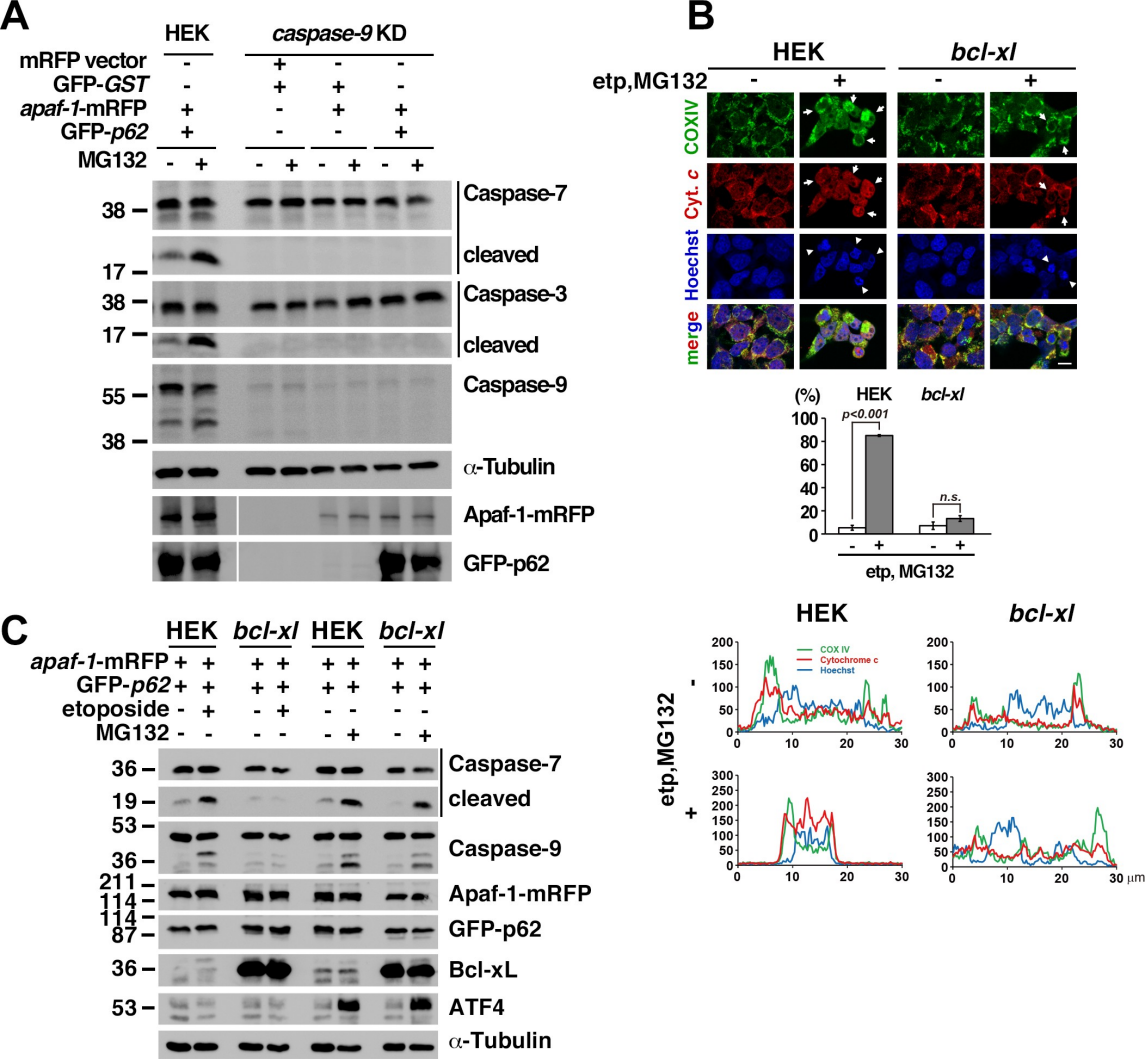
MG132 promotes caspase-9 activation in cells that stably express *bcl-xl*. (**A**) Caspase-3 and caspase-7 were activated upon MG132 treatment in cells expressing *apaf-1* and *p62* but not in *caspase-9* knockdown HEK293T cells (caspase-9 KD). HEK: parental HEK293T cells. (**B**) Wild type and *bcl-xl*-expressing stable HEK293T cells were treated with 100 μM etoposide for 32 h and 25 μM MG132 for 4 h. Localization of cytochrome *c* and COX IV (cytochrome *c* oxidase IV, mitochondrial marker) was visualized by immunocytochemistry. Release of cytochrome *c* from mitochondria was assessed by confocal microscope. Arrowheads and arrows represent nuclear fragmentation and cells, respectively. Scale bar: 10 μm. Graph shows percentage of cytochrome *c*-released cells. Lower right panels: the fluorescence intensity profile (arbitrary units) along the arrow is shown. (**C**) Caspase activation was examined in HEK293T cells stably expressing *bcl-xl* by treatment of etoposide for 32 h or MG132 for 4 h after transfection of *apaf-1* and *p62*. ATF4 was used as marker for proteasome inhibition, not for endoplasmic reticulum stress.

Western blotting revealed that the cleavage of both caspase-3 and caspase-7 was enhanced by treatment with MG132 in the presence of Apaf-1 and p62, which was not observed in *caspase-9* knockdown cells (Fig 4A). In response to apoptotic stimulation such as anticancer drug, UV irradiation, or DNA damage, cytochrome *c* is released from mitochondria to cytosol and makes apoptosome together with Apaf-1, procaspase-9, and deoxy ATP for caspase-9 activation[48]. Cytochrome *c* release is regulated by Bcl-2 family protein; Bax, Bak, and Bok promote and Bcl-2 and Bcl-xL prevent[49]. To examine the involvement of this cytochrome *c*–apoptosome pathway in our cell death, we used HEK293T cells that stably express *bcl-xl* to prevent mitochondrial cytochrome *c* release and downstream caspase-9 activation[49]. To examine property of these cells, we first treated with etoposide and MG132. Cytochrome *c* release was not observed in HEK293T cells stably expressing *bcl-xl*, but nuclear fragmentation was detected in these cells as well as in control HEK293 cells (arrows and arrowheads in Fig 4B). Colocalization of cytochrome *c* and COX IX in HEK293T cells stably expressing *bcl-xl* remained unchanged (Fig 4B). These data indicate that mitochondrial cytochrome *c* release was not involved in MG132-induced caspase-9 activation in our study. Then these cells were transfected with *apaf-1* and *p62* followed by treatment with etoposide, which suppressed the cleavage of caspase-9 and caspase-7 (Fig 4C). However, a 4-h treatment with MG132 resulted in caspase-9 and caspase-7 activation, as observed using Western blotting (Fig 4C). Because the mRNA expression of *bok* is already considerably low in HEK293T cells stably expressing *bcl-xl*, we further knocked down *bok* expression and found that MG132-induced caspase-9 activation was not further suppressed in these cells (S4B Fig). Bax levels were also not increased after a 4-h treatment with MG132 (S4A Fig).

**Fig 5:**
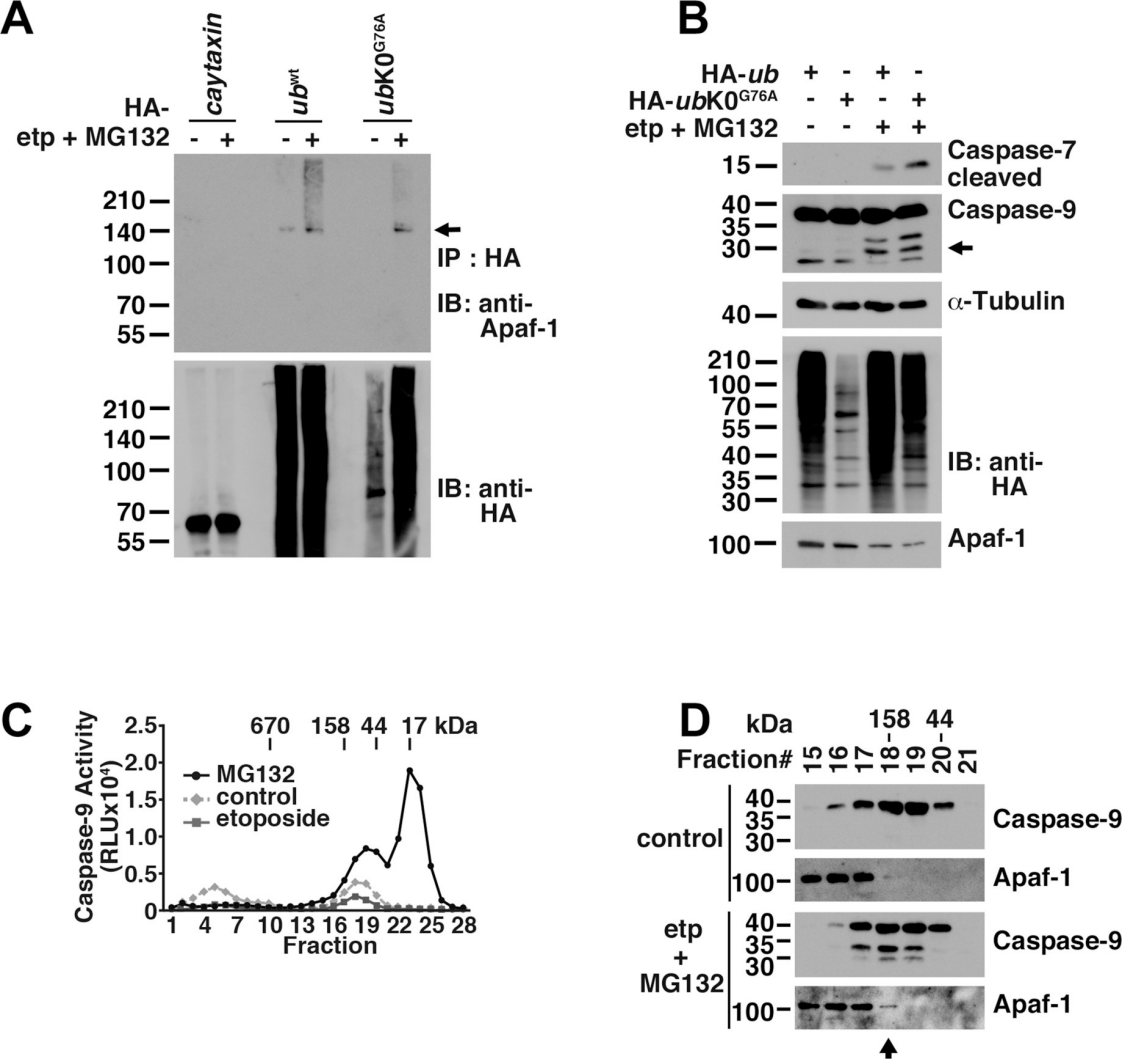
Ubiquitinated Apaf-1 interacts with p62 to activate caspase-9 in cells that stably express *bcl-xl*. (**A**) HEK293T cells stably expression *bcl-xl* were infected with *ubiquitin* wild type or dominant negative mutant (K0^G76A^), or transfected with Caytaxin as a negative control. HA-tagged ubiquitin and Caytaxin were precipitated with anti-HA-agarose antibody and ubiquitination was examined by western blot. (**B**) Caspase activation was examined in HEK293T cells stably expressing *bcl-xl* by treatment of etoposide for 32 h and MG132 for 4 h after infection of ubiquitin wild type or K0^G76A^ mutant. Arrow represented caspase-9 fragment. (**C**) *Apaf-1* and *p62* were transfected into *bcl-xl*-expressing HEK293T cells, and fractionation was performed following MG132 or etoposide treatment. Caspase-9 activity was determined using Caspase-Glo Assays. Similar result was obtained from three independent experiments. (**D**) HEK293T cells stably expressing *bcl-xl* were treated with etoposide for 32 h and MG132 for 4 h after infection of ubiquitin dominant negative mutant. Cell lysate was subjected to chromatography using Superose 6 10/300 GL column. Apaf-1 and caspase-9 were examined by western blot. Arrow represented free Apaf-1 protein in fraction #18.

To examine the effects of Apaf-1 ubiquitination on caspase-9 activation in *bcl-xl*-expressing cells, we infected wild type (wt) or *ub*K0^G76A^ mutant ubiquitin into HEK293T cells stably expressing *bcl-xl*, followed by treatment with etoposide and MG132. Although levels of the slower-migrating species of Apaf-1 were decreased in UbK0^G76A^-expressing cell lysates than in Ub^wt^-expressing cell lysates, the approximate 140 kDa Apaf-1 band that was precipitated by anti-ubiquitin antibodies was observed in both lysates (arrow in Fig 5A). In the presence of ubiquitinated Apaf-1 in UbK0^G76A^-expressing cells, caspase-7 and caspase-9 were both activated after treatment with etoposide and MG132 (Fig 5B). Although we did not determine the amount of ubiquitin that was conjugated to Apaf-1 in UbK0^G76A^-expressing cells, few molecules of ubiquitin on Apaf-1 are sufficient to form a complex with p62 and activate caspase-9 because p62 protein can bind to a single ubiquitin molecule *in vitro* [50]. To detect the cell fraction containing caspase-9 activity, we separated the S-100 extract (the 100,000 *× g* supernatant) using size-exclusion chromatography according to size with a Superose 6 10/300 GL column and performed a Caspase-Glo assay using *bcl-xl*-expressing HEK293T cell lysates infected with *apaf-1* and then treated with etoposide or MG132. Caspase-9 was activated in fractions with a mass smaller than 158 kDa from MG132-treated HEK293T cells compared with etoposide-treated HEK293T cells (Fig 5C and 5D). Ubiquitinated Apaf-1 fractions containing p62 with a mass smaller than 158 kDa might activate caspase-9 because p62 interacts with both ubiquitinated Apaf-1 and caspase-9 (Fig 3A), and MG132-induced caspase-7 and caspase-3 activation was suppressed in p62 knockdown cells (Fig 3D).

## Discussion

In this study, although Apaf-1 is degraded under normal conditions, treatment with etoposide stimulated the interaction between Apaf-1 and Cullin-4B, leading to Apaf-1 ubiquitination. Although the Apaf-1 homo-oligomer, called an apoptosome, is essential for caspase activation, results of the present study demonstrate that the complex comprising ubiquitinated Apaf-1 and p62 regulates caspase-9 activation under proteasome inhibition.

Cullin-4B is a member of the cullin-RING (a really interesting new gene) ubiquitin ligases that target WD40 repeat-containing proteins, such as WDR5[51] using damaged DNA binding protein complex (DDB) 1[52]; mutations of *cullin*-*4B* cause X-linked mental retardation (XLMR)[52]. Apaf-1 is a likely substrate of Cullin-4B because it has a WD40 repeat in its C-terminal region [3]. Additionally, treatment with etoposide enhances the interaction between Cullin-4B and Apaf-1 (Fig 2C), coincident with Apaf-1 ubiquitination (Fig 1G). Further, Apaf-1 expression is increased in the presence of siRNA for *cullin*-*4B* (Fig 2A), and its ubiquitination is decreased in *cullin-4B*-knockdown cells and increased in Cullin-4B-overexpressing cells (Fig 2D). These findings demonstrate that Cullin-4B targets Apaf-1 ubiquitination. Interestingly, the number of apoptotic cells are increased in *cullin-4B*-knockout mice embryos[53, 54], and there are no cases of cancer susceptibility to XLMR linked to *cullin-4B* mutations [55]. Because Apaf-1 expression was increased in HEK293T cells expressing siRNA for *cullin-4B* or dominant-negative *cullin-4B* (Fig 2A and 2B), the apoptosome may activate caspase-9 in *cullin-4B*-knockout mice and in cases of XLMR; however, more experiments are needed.

Using a systematic screen, the lysine at position 224 of human Apaf-1 was identified as a ubiquitination site in bortezomib-treated cells[43]. Because Apaf-1 4KR (K192, 224, 252, and 637R) ubiquitination was decreased in HEK293T cells (S1E Fig), the lysine at position 224 of human Apaf-1 may be a site of ubiquitination. Similarly, CED-4 K212R ubiquitination was decreased in HEK293T cells (S1D Fig). A lysine at position 212 of CED-4 was generated by alternate splicing of the extended region of exon 4 found in *ced-4L*[5]. Interestingly, the *ced-4 (n2273)* mutation, which has an arginine-to-lysine substitution at position 236 of CED-4L, results in enhanced apoptosis from the *ced-9 (n1653)* allele [56], although it is unknown whether CED-4 ubiquitination regulates CED-3 activity. Caspase-8 is ubiquitinated by Cullin-3 following apoptotic stimulation by TRAIL, leading to p62-dependent aggregation and caspase-8 activation [33]. Similarly, after treatment with etoposide, Apaf-1 is subjected to Cullin-4B-dependent ubiquitination, which forms aggregates in a p62 UBA domain-dependent manner and activates caspase-9 following treatment with MG132. Caspase-8 ubiquitination by Cullin-3 mediates the positively extrinsic apoptotic pathway, whereas Apaf-1 ubiquitination by Cullin-4B enhances the intrinsic apoptotic pathway under proteasome inhibition. However, Apaf-1 is quickly degraded under normal conditions (Fig 1B), suggesting that the intrinsic apoptotic pathway is negatively regulated in healthy cells. Consistent with these findings, Cullin-4B has been shown to promote cell proliferation and inhibit apoptosis[57].

The localization of CED-4 may be important for both apoptosis[19] and CED-3 expression[18]; however, the site of Apaf-1 aggregation during the process of caspase-9 activation is unclear. Using a combination of fluorescence and electron microscopy, Apaf-1 aggregates were found in the cytosol comprising membrane structures such as the rough ER and mitochondria (S3A Fig). Previous reports have shown that the aggregates, which are formed in a p62-dependent manner, accumulate rough ER and ubiquitinated proteins in *Atg7*-deficient hepatocytes and that liver injury is rescued by a loss of p62[46]. We also found that Apaf-1 aggregates and MG132-induced caspase-3 and caspase-7 activation were both dependent on p62 (Fig 3C and S3B Fig). Using chromatography, high levels of free Apaf-1 were detected in caspase-9-activated fraction #18 (lower panel in Fig 5D). These results demonstrate that free Apaf-1 might activate caspase-9 in the cytosol.

Several reports have described the mechanisms by which proteasome inhibitors can induce apoptosis[49, 58–62] (S5 Fig). Zhu et al. showed that MG132 potentiates caspase-8 activation via DR5 upregulation[62]. Herrmann et al. showed that acetyl-leucinal-leucinal-nor-leucinal (ALLnL) and acetyl-leucinal-leucinal-nor-methional (ALLM) both induce apoptosis via prostate apoptosis response-4 (PAR-4) upregulation independent of Bcl-2[59]. An et al. showed that the dipeptidyl proteasome inhibitor CEP1612 induces apoptosis via YVAD-inhibited caspase activation [61], which may occur via caspase-1[63]. Henderson et al. showed that MG132 stabilizes Smac to activate caspases via Bcl-2 inhibition independent of caspase-9[60]. Liambi et al. showed that Bok is stabilized by proteasome inhibitors and induces mitochondrial apoptosis independent of Bax and Bak[49]. Interestingly, Almond et al. suggested that proteasome inhibition activates caspases via an alternative mechanism that does not involve apoptosome formation [64]. In our study, MG132-induced apoptosis was not inhibited by Bcl-xL but was dependent on caspase-9. Bax expression was not increased (S4A Fig), and cytochrome *c* release was not observed. These results suggest that the sequential application of apoptotic stimuli and proteasome inhibition results in enhanced programmed cell death via caspase-9 activation by the alternative Apaf-1 complex, which includes Apaf-1, ubiquitin, and p62 (S5 Fig).

## Supporting information

S1 Fig Apaf-1 homologs CED-4 and Dapaf-1 are ubiquitinated in mammalian cells. (**A**) Immunoprecipitation was performed using anti-HA-agarose or anti-FLAG M2 affinity gel after transfection of HEK293T cells with *apaf-1*-FLAG and HA-*ubiquitin*. IP: immunoprecipitation; IB: immunoblot; Ub: ubiquitin; wcl: whole cell lysate. (**B**, **C**) Ubiquitinated Dapaf-1 (b) and CED-4 (c) were accumulated following MG132 treatment. (**D**) Level of ubiquitination of CED-4 and CED-4L. S: CED-4; L: CED-4L. The level of ubiquitination was corrected using immunoprecipitated CED-4 (IB: anti-HA). Graphs represent mean and independent data points from three independent experiments. (**E**) Ubiquitination assay using Apaf-1 4KR mutant following etoposide treatment. Graphs represent mean and independent data points from three independent experiments. (**F**) Apaf-1 was ubiquitinated in untreated *cullin-4B*-knocked down cells.
(PDF)

S2 Fig Alignment of α/β-fold domain of Apaf-1 and CED-4. (**A**) Apaf-1 sequences are from human (NM_181861), cow (NM_001191507), dog (XM_861410), gorilla (XM_004053729), zebrafish (NM_131608), *Drosophila* (AB027531), and *C*. *elegans* CED-4L *n2273* and CED-4S sequences are from NM_001026031. Amino acids are indicated by single letter code. (**B**) Lysine-to-arginine substitution around the region with 24 amino acid insertion in *ced-4L* (upper panel). *ced-4L n2273* mutation results in arginine-to-lysine substitution (arrow). Lysine-to-arginine substitution in Apaf-1 (lower panel).
(PDF)

S3 Fig Localization of Apaf-1 aggregate in the cells. (**A**) A combination of fluorescence and electron microscopy was performed after the transfection of HEK293T cells with *apaf-1*-mRFP and GFP-*p62*. Top panels: images of confocal and electron microscopy. Scale bar: 10 μm. Bottom panels: arrow and arrowheads show ER and mitochondria, respectively. The left, middle, and right panels show higher magnification images of the rectangles from the top EM panel, left panel, and middle panel, marked rectangle, respectively. Scale bar: 5 μm (left panel); 2 μm (middle panel); 500 nm (right panel). (**B**) Apaf-1-mRFP forms aggregates in the cytosol upon GFP-*p62* co-transfection in HEK293T cells (left panels). (-): no treatment; (+): MG132 treatment. The size of aggregates showed an increase with MG132 treatment (right panel). (**C**) Co-immunoprecipitation was performed following the transfection of *apaf-1*-HA together with FLAG-*p62* or FLAG-del UBA p62 into HEK293T cells. Apaf-1 interacted with full length and del UBA p62. (**D**) Expression of full length p62, but not del UBA p62, activated caspase-3, caspase-7, and caspase-9.
(PDF)

S4 Fig Etoposide and MG132 induced caspase-9 activation. (**A**) The expression of apoptosis-related proteins in whole cell lysate for ubiquitination assay shown in Fig 5A. (**B**) *Bok* mRNA expression in *bok* knockdown HEK293T cells stably expressing *bcl-xl*. Top; relative expression of *bok* mRNA in three cell lines examined by real time RT-PCR, middle; relative levels of *bok* mRNA in knockdown HEK293T cells stably expressing *bcl-xl* analyzed by semi quantitative RT-PCR, bottom; Caspase-9 activation by treatment of etoposide and MG132. (**C**) HEK293T cells stably expressing *bcl-xl* and ubiquitin or p62-knocked down cells were treated with etoposide for 32 h and MG132 for 4 h. Cell lysate was subjected to chromatography using Superose 6 10/300 GL column. p62 was examined by western blot. Arrow represented p62 protein in fraction #18.
(PDF)

S5 Fig Schematic cascade of proteasome inhibitor-induced apoptosis. (PDF)
