# Supplementary figures and images for: Cullin-4B E3 ubiquitin ligase mediates Apaf-1 ubiquitination to regulate caspase-9 activity
Source: PLoS One. 2019 Jul 22;14(7):e0219782. doi: 10.1371/journal.pone.0219782 (PMC6645535; doi:10.1371/journal.pone.0219782)

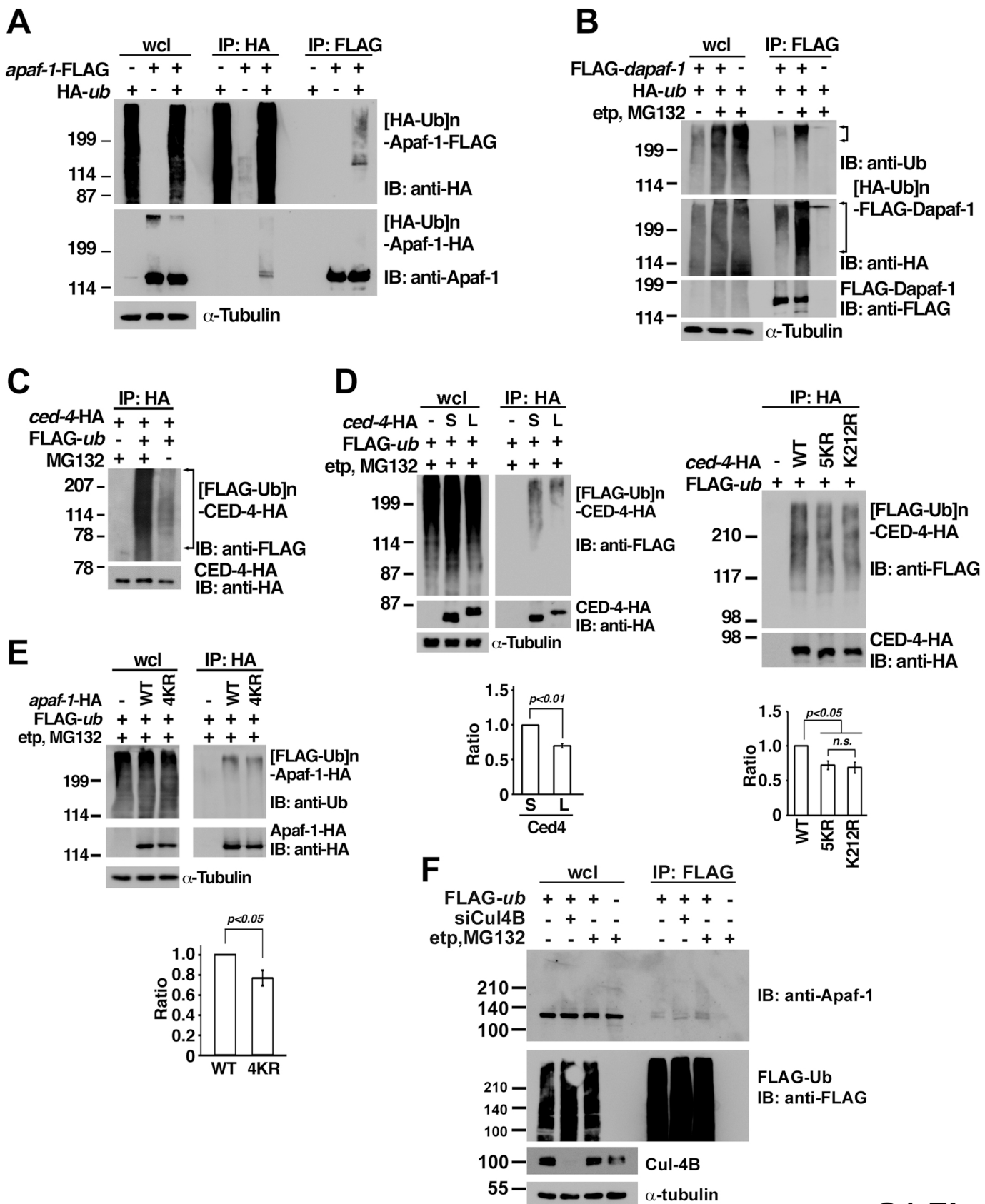

S1 Fig

Supplement: S1 Fig — (A) Immunoprecipitation was performed using anti-HA-agarose or anti-FLAG M2 affinity gel after transfection of HEK293T cells with apaf-1-FLAG and HA-ubiquitin. IP: immunoprecipitation; IB: immunoblot; Ub: ubiquitin; wcl: whole cell lysate. (B, C) Ubiquitinated Dapaf-1 (b) and CED-4 (c) were accumulated following MG132 treatment. (D) Level of ubiquitination of CED-4 and CED-4L. S: CED-4; L: CED-4L. The level of ubiquitination was corrected using immunoprecipitated CED-4 (IB: anti-HA). Graphs represent mean and independent data points from three independent experiments. (E) Ubiquitination assay using Apaf-1 4KR mutant following etoposide treatment. Graphs represent mean and independent data points from three independent experiments. (F) Apaf-1 was ubiquitinated in untreated cullin-4B-knocked down cells. (PDF) [file pone.0219782.s001.pdf]

**A**

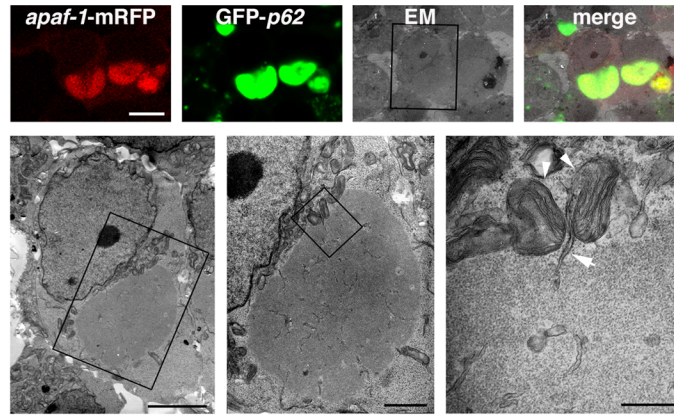

**B**

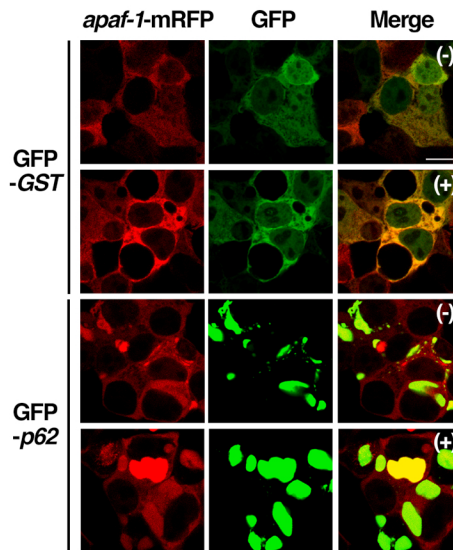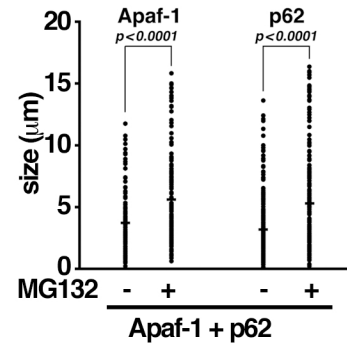

**C**

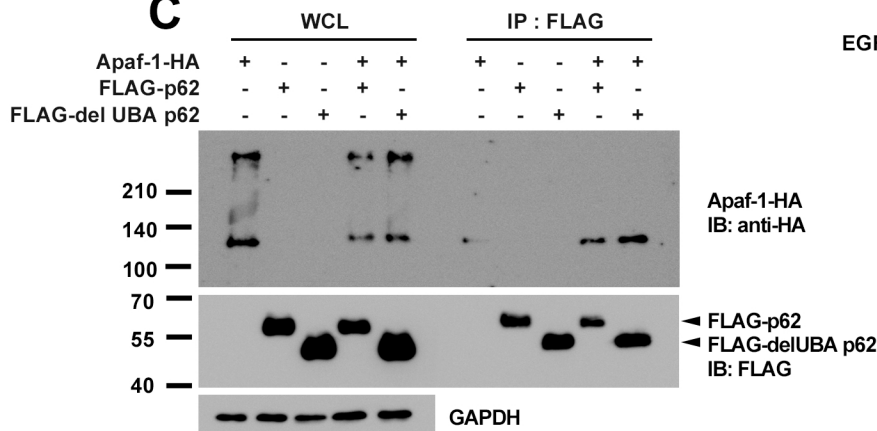

**D**

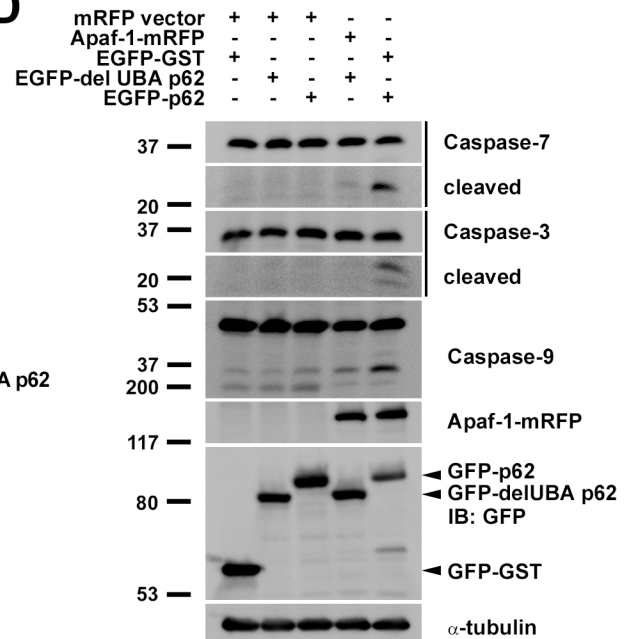

**S3 Fig**

Supplement: S3 Fig — (A) A combination of fluorescence and electron microscopy was performed after the transfection of HEK293T cells with apaf-1-mRFP and GFP-p62. Top panels: images of confocal and electron microscopy. Scale bar: 10 μm. Bottom panels: arrow and arrowheads show ER and mitochondria, respectively. The left, middle, and right panels show higher magnification images of the rectangles from the top EM panel, left panel, and middle panel, marked rectangle, respectively. Scale bar: 5 μm (left panel); 2 μm (middle panel); 500 nm (right panel). (B) Apaf-1-mRFP forms aggregates in the cytosol upon GFP-p62 co-transfection in HEK293T cells (left panels). (-): no treatment; (+): MG132 treatment. The size of aggregates showed an increase with MG132 treatment (right panel). (C) Co-immunoprecipitation was performed following the transfection of apaf-1-HA together with FLAG-p62 or FLAG-del UBA p62 into HEK293T cells. Apaf-1 interacted with full length and del UBA p62. (D) Expression of full length p62, but not del UBA p62, activated caspase-3, caspase-7, and caspase-9. (PDF) [file pone.0219782.s003.pdf]

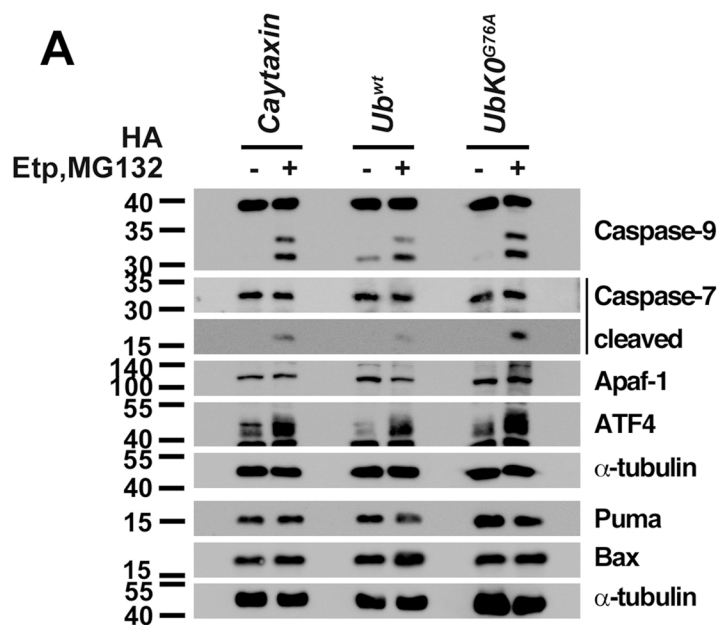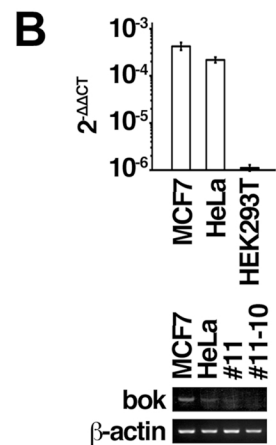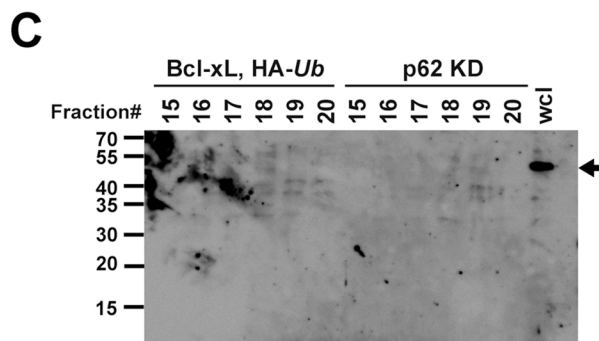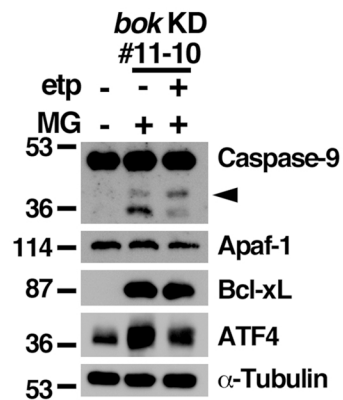

Supplement: S4 Fig — (A) The expression of apoptosis-related proteins in whole cell lysate for ubiquitination assay shown in Fig 5A. (B) Bok mRNA expression in bok knockdown HEK293T cells stably expressing bcl-xl. Top; relative expression of bok mRNA in three cell lines examined by real time RT-PCR, middle; relative levels of bok mRNA in knockdown HEK293T cells stably expressing bcl-xl analyzed by semi quantitative RT-PCR, bottom; Caspase-9 activation by treatment of etoposide and MG132. (C) HEK293T cells stably expressing bcl-xl and ubiquitin or p62-knocked down cells were treated with etoposide for 32 h and MG132 for 4 h. Cell lysate was subjected to chromatography using Superose 6 10/300 GL column. p62 was examined by western blot. Arrow represented p62 protein in fraction #18. (PDF) [file pone.0219782.s004.pdf]

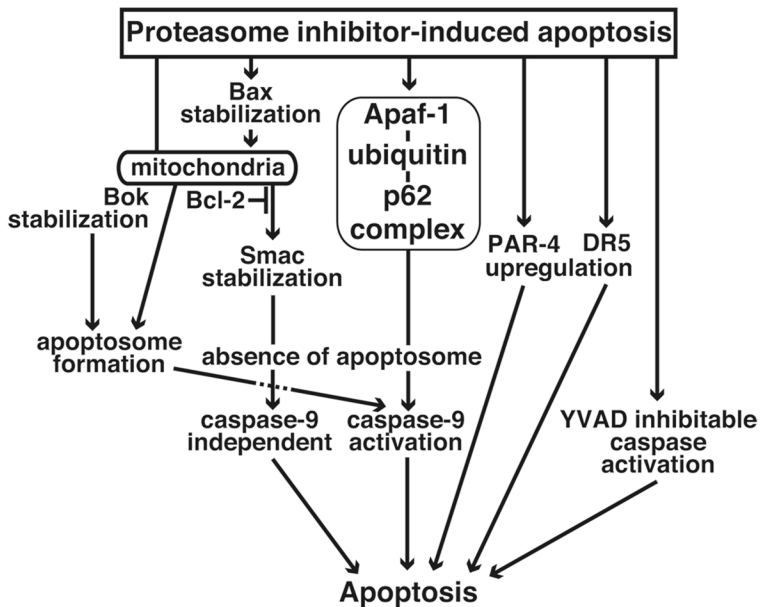

**S5 Figure**

Supplement: S5 Fig — (PDF) [file pone.0219782.s005.pdf]
