# Supplementary material for: Cullin-4B E3 ubiquitin ligase mediates Apaf-1 ubiquitination to regulate caspase-9 activity
Source: PLoS One. 2019 Jul 22;14(7):e0219782. doi: 10.1371/journal.pone.0219782 (PMC6645535; doi:10.1371/journal.pone.0219782)
Supplement: S2 Fig — (A) Apaf-1 sequences are from human (NM_181861), cow (NM_001191507), dog (XM_861410), gorilla (XM_004053729), zebrafish (NM_131608), Drosophila (AB027531), and C. elegans CED-4L n2273 and CED-4S sequences are from NM_001026031. Amino acids are indicated by single letter code. (B) Lysine-to-arginine substitution around the region with 24 amino acid insertion in ced-4L (upper panel). ced-4L n2273 mutation results in arginine-to-lysine substitution (arrow). Lysine-to-arginine substitution in Apaf-1 (lower panel). (PDF) [file pone.0219782.s002.pdf]

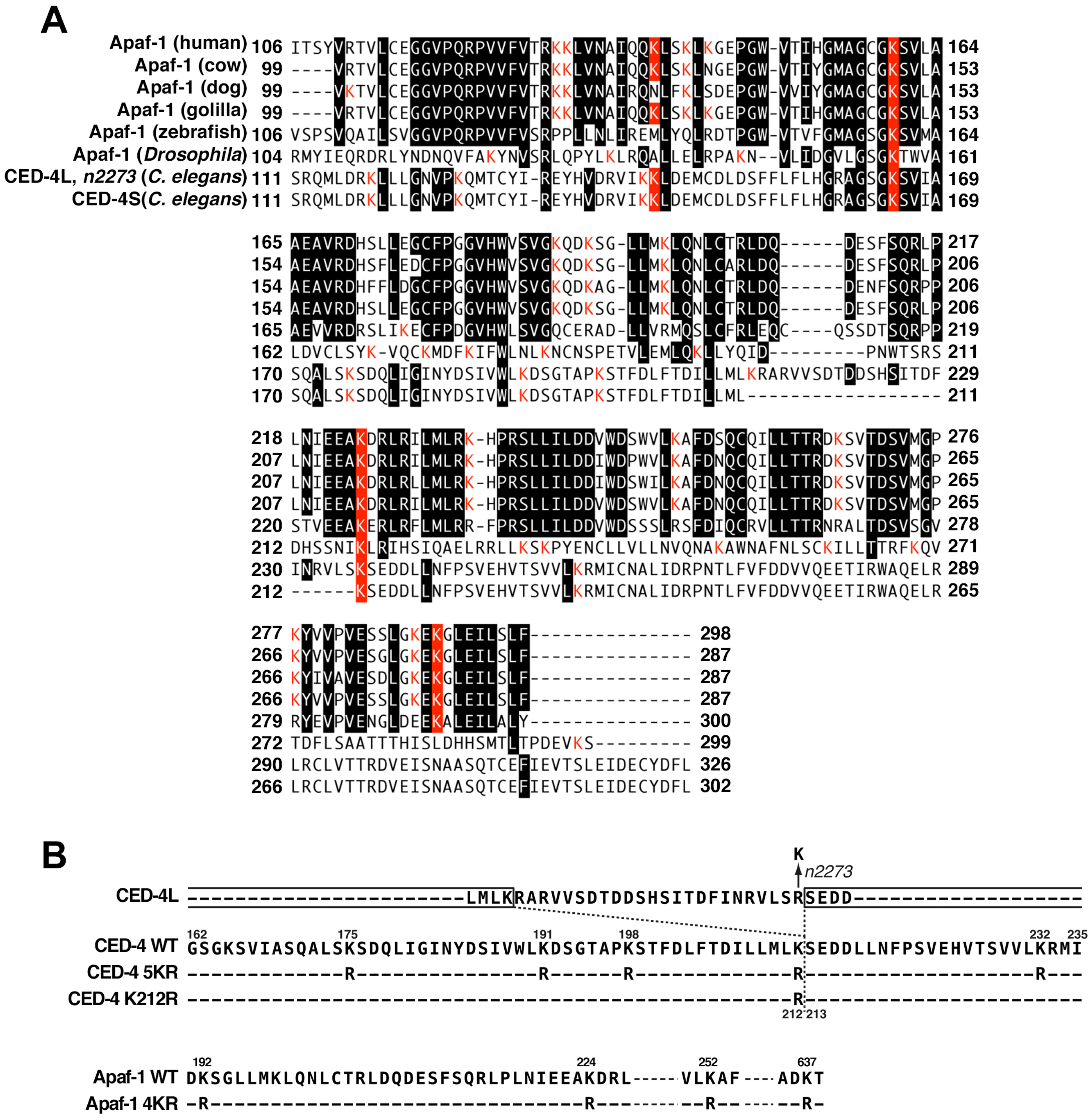
A
Apaf-1 (human) 106 ITSYVRTVLCEGGVPQRPVVFVTRKKLVNAIQQKLSKLKGEPGW-VTIHGMAGCGKSVLA 164
Apaf-1 (cow) 99 ----VRTVLCEGGVPQRPVVFVTRKKLVNAIQQKLSKLNGEPGW-VTIYGMAGCGKSVLA 153
Apaf-1 (dog) 99 ----VKTVLCEGGVPQRPVVFVTRKKLVNAIRQNLFKLSDEPGW-VVIYGMAGCGKSVLA 153
Apaf-1 (golilla) 99 ----VRTVLCEGGVPQRPVVFVTRKKLVNAIQQKLSKLKGEPGW-VTIHGMAGCGKSVLA 153
Apaf-1 (zebrafish) 106 VSPSVQAILSVGGVPQRPVVFVSRPPLLNLIREMLYQLRDTPGW-VTVFGMAGSGKSVMA 164
Apaf-1 (Drosophila) 104 RMYIEQRDRLYNDNQVFAKYNVSRLQPYLKLRQALLELRPAKN--VLIDGVLGSGKTWVA 161
CED-4L, n2273 (C. elegans) 111 SRQMLDRKLLLGNVPKQMTCYI-REYHVDRVIKKLDEMCDLDSFFLFLHGRAGSGKSVIA 169
CED-4S(C. elegans) 111 SRQMLDRKLLLGNVPKQMTCYI-REYHVDRVIKKLDEMCDLDSFFLFLHGRAGSGKSVIA 169
165 AEAVRDHSLLEGCFPGGVHWVSVGKQDKSG-LLMKLQNLCTRLDQ------DESFSQRLP 217
154 AEAVRDHSFLEDCFPGGVHWVSVGKQDKSG-LLMKLQNLCARLDQ------DESFSQRLP 206
154 AEAVRDHFFLDGCFPGGVHWVSVGKQDKAG-LLMKLQNLCTRLDQ------DENFSQRPP 206
154 AEAVRDHSLLEGCFPGGVHWVSVGKQDKSG-LLMKLQNLCTRLDQ------DESFSQRLP 206
165 AEVVRDRSLIKECFPDGVHWLSVGQCERAD-LLVRMQSLCFRLEQC----QSSDTSQRPP 219
162 LDVCLSYK-VQCKMDFKIFWLNLKNCNSPETVLEMLQKLLYQID---------PNWTSRS 211
170 SQALSKSDQLIGINYDSIVWLKDSGTAPKSTFDLFTDILLMLKRARVVSDTDDSHSITDF 229
170 SQALSKSDQLIGINYDSIVWLKDSGTAPKSTFDLFTDILLML------------------ 211
218 LNIEEAKDRLRILMLRK-HPRSLLILDDVWDSWVLKAFDSQCQILLTTRDKSVTDSVMGP 276
207 LNIEEAKDRLRILMLRK-HPRSLLILDDIWDPWVLKAFDNQCQILLTTRDKSVTDSVMGP 265
207 LNIEEAKDRLRLLMLRK-HPRSLLILDDIWDSWILKAFDNQCQILLTTRDKSVTDSVMGP 265
207 LNIEEAKDRLRILMLRK-HPRSLLILDDVWDSWVLKAFDNQCQILLTTRDKSVTDSVMGP 265
220 STVEEAKERLRFLMLRR-FPRSLLILDDVWDSSSLRSFDIQCRVLLTTRNRALTDSVSGV 278
212 DHSSNIKLRIHSIQAELRRLLKSKPYENCLLVLLNVQNAKAWNAFNLSCKILLTTRFKQV 271
230 INRVLSKSEDDLLNFPSVEHVTSVVLKRMICNALIDRPNTLFVFDDVVQEETIRWAQELR 289
212 ------KSEDDLLNFPSVEHVTSVVLKRMICNALIDRPNTLFVFDDVVQEETIRWAQELR 265
277 KYVVPVESSLGKEKGLEILSLF--------------- 298
266 KYVVPVESGLGKEKGLEILSLF--------------- 287
266 KYIVAVESDLGKEKGLEILSLF--------------- 287
266 KYVVPVESSLGKEKGLEILSLF--------------- 287
279 RYEVPVENGLDEEKALEILALY--------------- 300
272 TDFLSAATTTHISLDHHSMTLTPDEVKS--------- 299
290 LRCLVTTRDVEISNAASQTCEFIEVTSLEIDECYDFL 326
266 LRCLVTTRDVEISNAASQTCEFIEVTSLEIDECYDFL 302
B
K
n2273
CED-4L ----------------------LMLKRARVVSDTDDSHSITDFINRVLSRSEDD--------------------
162 175 191 198 232 235
CED-4 WT GSGKSVIASQALSKSDQLIGINYDSIVWLKDSGTAPKSTFDLFTDILLMLKSEDDLLNFPSVEHVTSVVLKRMI
CED-4 5KR -------------R---------------R------R-------------R--------------------R---
CED-4 K212R --R------------------------
212 213
192 224 252 637
Apaf-1 WT DKSGLLMKLQNLCTRLDQDESFSQRLPLNIEEAKDRL----- VLKAF ---- ADKT
Apaf-1 4KR -R------------------------------R--------- --R-- ---- --R-

S2 Fig
